# Use of Smartphones and Wrist-Worn Devices for Motor Symptoms in Parkinson’s Disease: A Systematic Review of Commercially Available Technologies

**DOI:** 10.3390/s25123732

**Published:** 2025-06-14

**Authors:** Gabriele Triolo, Daniela Ivaldi, Roberta Lombardo, Angelo Quartarone, Viviana Lo Buono

**Affiliations:** IRCCS Centro Neurolesi Bonino Pulejo, 98124 Messina, Italy; gabriele.triolo@irccsme.it (G.T.); roberta.lombardo@irccsme.it (R.L.); angelo.quartarone@irccsme.it (A.Q.); viviana.lobuono@irccsme.it (V.L.B.)

**Keywords:** Parkinson’s Disease, mHealth, smartphone, smartwatch, activity trackers, UPDRS, usability

## Abstract

Parkinson’s disease (PD) is a progressive neurodegenerative disorder characterized by motor symptoms such as tremors, rigidity, and bradykinesia. The accurate and continuous monitoring of these symptoms is essential for optimizing treatment strategies and improving patient outcomes. Traditionally, clinical assessments have relied on scales and methods that often lack the ability for continuous, real-time monitoring and can be subject to interpretation bias. Recent advancements in wearable technologies, such as smartphones, smartwatches, and activity trackers (ATs), present a promising alternative for more consistent and objective monitoring. This review aims to evaluate the use of smartphones and smart wrist devices, like smartwatches and activity trackers, in the management of PD, assessing their effectiveness in symptom evaluation and monitoring and physical performance improvement. Studies were identified by searching in PubMed, Scopus, Web of Science, and Cochrane Library. Only 13 studies of 1027 were included in our review. Smartphones, smartwatches, and activity trackers showed a growing potential in the assessment, monitoring, and improvement of motor symptoms in people with PD, compared to clinical scales and research-grade sensors. Their relatively low cost, accessibility, and usability support their integration into real-world clinical practice and exhibit validity to support PD management.

## 1. Introduction

Parkinson’s disease (PD) is a progressive neurological disorder characterized by a wide range of motor and non-motor features which can impact daily functioning to varying degrees [1]. The hallmark motor symptoms of PD include bradykinesia, tremor, rigidity, and gait disturbances, such as shuffling steps and postural instability, which increase also the risk of falls [2,3]. The early detection of PD is crucial for slowing disease progression and maximizing the benefits of disease-modifying therapies, which are most effective when initiated in the early stages [4,5]. In addition, the management of PD requires continuous symptom monitoring and the implementation of personalized treatment strategies to optimize clinical outcomes.

In this context, to achieve an effective result, tools capable of accurately and continuously monitoring symptoms are essential.

Clinical assessment tools, such as the MDS-UPDRS (a revised version of the Unified Parkinson’s Disease Rating Scale) that assesses non-motor, motor, and motor complications in PD [6], have demonstrated strong reliability in monitoring within-subject changes in disease progression under real-world conditions [7]. Especially, the MDS-UPDRS part II, focused on ADL, can be used to assess daily living in Parkinson’s, while part III is the worldwide reference scale to evaluate Parkinsonian motor impairment, especially bradykinesia [8,9]. Similarly, the Hoehn & Yahr scale defines broad categories of motor function in PD, resulting in a widely used tool that provides a simple and effective way to classify motor impairment severity, correlating with disease progression, quality of life, and neuroimaging findings [10]. Over the past decade, a wide range of technology-based objective measures for assessing impairments have been developed, offering the potential to significantly transform the diagnostic, monitoring, and therapeutic landscape of PD. Among these, wearable sensor-based technologies are transforming PD care by enabling the continuous, unobtrusive, and objective monitoring of motor symptoms. These advancements not only enhance the accuracy of diagnosis and treatment assessment but also provide ecologically valid data that can improve patient–doctor interactions and inform therapeutic decisions [11,12]. Furthermore, wearable technologies, like smartwatches or activity trackers (ATs) and mobile health (mHealth) applications provided by smartphones, offer a promising approach for symptom assessment. These devices enable the objective, high-resolution, and continuous monitoring of motor impairments, providing valuable data for both clinical management and research. In particular, smartphones equipped with embedded sensors and advanced communication technologies facilitate remote and continuous health monitoring at minimal additional cost [13,14]. Smartphones, smartwatches, and ATs, also even low-cost ones, represent accessible and cost-effective solutions that can facilitate real-time health tracking and provide valuable clinical insights, due to their usability that can increase acceptability and user satisfaction [15].

Owing to the widespread adoption of portable multimedia devices, smartphone usage has become ubiquitous worldwide. In recent years, the global number of smartphone users has been estimated to exceed 3.8 billion, representing a twofold increase compared to a decade ago [16]. Although smartphones are often mistaken for wearable devices, they do not fully meet the definition of wearables. The key features of wearable health devices include customization, wearability, skin-conformity, long-term monitoring capability, and sustainable power sources [17]. However, smartphones could still function as wearable-like tools when secured with straps, carried in a pocket, or used for video-based movement analysis. These alternative methods of use allow smartphones to capture relevant motor data, making them a versatile option for assessing movement disorders despite their limitations in continuous monitoring.

Wrist-worn smart devices function as wearable devices. Depending on their specific features, these devices are categorized as smartwatches, smart bracelets, wristbands, or ATs. The development of smartwatches within the consumer market reflects the rapid technological advancements of the past three decades, particularly in the miniaturization of electronic devices. While early smartwatches were introduced before 2012 without sophisticated operating systems, the release of more advanced models in 2012 marked a turning point, enabling these devices to be effectively used for health monitoring and symptom tracking [18].

In the management of PD, these devices can support both clinicians and patients by enabling the continuous monitoring of motor symptoms, offering immediate feedback, and potentially improving treatment adherence. In addition, the integration of wearable technologies into clinical care enables remote health monitoring, making it possible for patients to receive personalized care, reducing the need for frequent outpatient visits. This approach enhances care convenience, reduces healthcare system burden, and improves resource efficiency. Real-time tracking allows for the early detection of motor and non-motor fluctuations, enabling timely adjustments in therapy. This ongoing monitoring not only improves clinical decision-making but also enhances patient outcomes by providing a more personalized approach to care. Furthermore, continuous symptom tracking helps to identify subtle changes that may not be captured during traditional clinic visits, ultimately contributing to a more comprehensive understanding of the disease.

The aim of this review is to explore the use of smartphones, smartwatches, and ATs in assessing and monitoring motor symptoms and improving physical performance in pwPD (people with Parkinson’s disease) by using sensors embedded in these commercially available devices, investigating their usability in clinical practice, their promiscuous or stand-alone use, and their capacity to be validated against other standards (e.g., traditional assessment, validated wearable sensors, healthy subjects).

## 2. Materials and Methods

This systematic review was conducted and reported in accordance with the Preferred Reporting Items for Systematic Review and Meta-Analyses (PRISMA). The studies were identified by searching in PubMed, Scopus, Web of Science, and Cochrane Library databases between February and March 2025. The PICO framework was used to formulate our research question and assist the study selection process [19]. Our target population comprised adults (>18 years) affected by PD. The intervention involved the use of a smartphone, smartwatch, or wrist-worn commercial device to assess, monitor, or improve motor symptoms. For comparison, we considered other sensors or traditional assessment methods. The primary outcome focused on the technical validity of commercially available smartphones, smartwatches, or ATs in evaluating, monitoring, and improving motor symptoms; a secondary outcome was to investigate the clinical usability and acceptance of these devices, as reported in the selected studies. All the studies fulfilling our selected criteria were evaluated for possible inclusion.

The search queries were designed to combine terms related to mobile devices (smartphones, smartwatches, wristbands, wrist devices) with key concepts associated with PD and their application in symptom assessment and monitoring. The final search combined the following terms for each database:

- ((smartphone OR smartwatch OR wristband OR wrist device) AND (digital health OR rehabilitation OR motor outcome OR fall OR gait)) AND (parkinson).

Two investigators (G.T. and D.I.) independently screened the articles in a three-step process: title screening, abstract screening, and full-text review. Any disagreements regarding study selection were resolved through discussion, with a third reviewer (V.L.B.) consulted when necessary.

**Figure 1:**
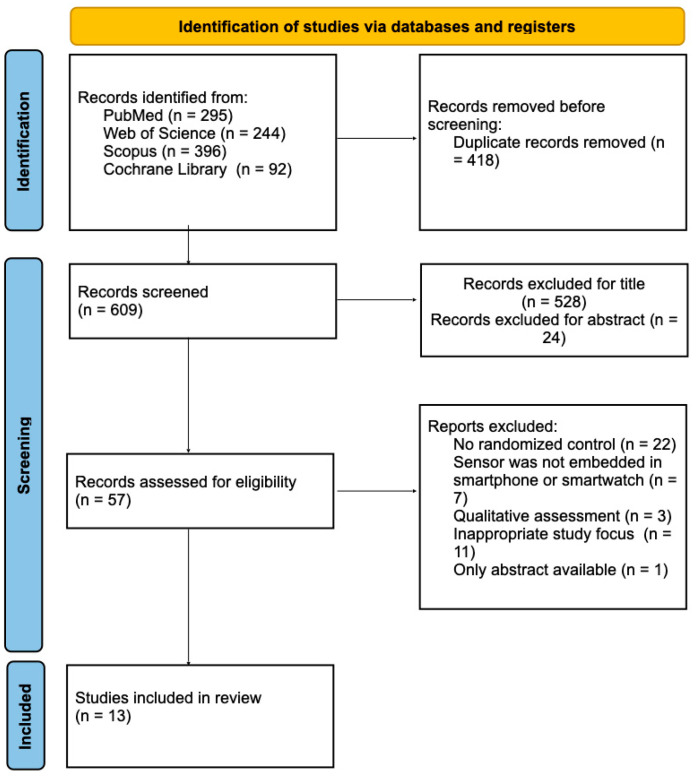
PRISMA flow chart.

A total of 1027 records were initially identified. After removing duplicates, 609 records underwent title screening (Figure 1).

A total of 81 articles assessed for eligibility and were screened by abstract. The list of articles was then refined for relevance, revised, and summarized, with key topics identified based on the inclusion/exclusion criteria.

### 2.1. Inclusion Criteria

Studies were included if they met the following criteria:

- Original articles;
- Studies that used quantitative assessment;
- Studies that included individuals diagnosed with PD as the experimental group;
- Studies that studied the use of a smartphone or smartwatch or both for monitoring or assessing motor symptoms in PD compared with the control group;
- Studies that reported outcomes based on data collected from embedded sensors within smartphones, smartwatches, or ATs;
- Studies that used smartphones, smartwatches, or ATs to assess or monitor motor symptoms or to improve motor performance;
- Studies that presented results from studies where data were collected using humans;
- English articles.

### 2.2. Exclusion Criteria

Studies were excluded if they met the following criteria:

- Qualitative studies;
- Studies that investigated only medication response or pharmacological treatments,
- Studies that evaluated algorithmic approaches or artificial intelligence;
- Studies that relied exclusively on self-reported diary data as the primary outcome for clinical evaluation;
- Studies that involved a direct intervention by a therapist;
- Studies involving other pathologies.
- Finally, after full-text evaluation, only 13 studies were included in this review.

## 3. Results

A total of 1027 articles were identified through searches of the PubMed, Cochrane, Web of Science, and Scopus databases. Of the articles identified, 418 duplicate articles were deleted; 528 studies were excluded after title screening and 24 were excluded after abstract screening; and 57 studies underwent full-article screening to assess eligibility. Finally, only 13 articles met the inclusion criteria (see Table 1).

The reviewed studies provide valuable insights into how smartphones, smartwatches, and AT, either individually or in combination, can improve healthcare management in pwPD. The number of participants across the included studies ranged from 20 to 132. Monitoring duration varied substantially, from single-session laboratory assessments to continuous home-based tracking over a 12-month period. Devices were worn on various anatomical locations, including the wrist, hip, and lower back, depending on the study protocol. These variations highlight both the versatility and heterogeneity of commercially available technologies for tracking motor symptoms in PD.

Four studies evaluated the use of smartphones in combination with smartwatches to collect data or monitor symptoms in pwPD [26,27,30,31]. Eight studies evaluated the use of smartphones as stand-alone devices to assess, monitor, or improve motor performances in pwPD [20,21,22,23,24,25,28,29]. No study specifically evaluated smartwatches independently, and only one study assessed standalone AT for step counting [32].

The studies used active tasks (e.g., tapping tests, TUG, and motor exercises) [20,23,24,25,28,29,30] or passive monitoring, like accelerometry data during daily activities, to track motor symptoms such as bradykinesia, tremor, gait disturbances, postural instability, and dexterity [21,22,26,27,31,32].

To provide a clear and rapid overview of the main aspects investigated by each study included in this review, a summary table was developed (see Table 2).

### 3.1. Correlation with Clinical Measures or Wearable Sensors

There is a strong correlation between digital assessments and established clinical scales, like the UPDRS or TUG test. Two studies demonstrated that smartphone applications accurately reflected clinical assessments of bradykinesia and gait disturbances [20,22]. Data from the combined use of smartphones and smartwatches have been shown to correlate significantly with clinical evaluations of resting tremor and bradykinesia, suggesting their potential for clinical application and remote monitoring [31]. A smartphone application using selected items from the UPDRS has demonstrated utility in predicting disease severity, supporting its use in monitoring disease progression [23]. In line with other findings supporting the clinical validity of wearable-derived metrics, data derived from the smartphone-based TUG may serve as objective measures for assessing motor characteristics and fall risk in PD and show significant associations with specific parameters of both the UPDRS [24]. In addition, commercially available smartwatches and smartphone applications have been shown to accurately capture motor and non-motor features of early, untreated PD, correlating strongly with the MDS-UPDRS [27].

Furthermore, four studies compared results from smartphone or smartwatch data to validated wearable sensors, reporting positive correlations that support the clinical utility of these technologies. One study compared a smartphone gait assessment to a heelmounted footswitch sensors and a pressure sensor walkway (GAITrite) [22]. In [32], two commercially available ATs were compared to a validated research-grade sensor, Dynaport Movemonitor (DAM), and [27] compared measurement from a smartphone and smartwatch to a research-grade wearable sensor (OPAL).

### 3.2. Longitudinal Monitoring of Symptoms and Treatment

The included studies varied in terms of monitoring length, with several reporting the duration of either symptom tracking or intervention delivery, ranging from 14 days to 12 months [23,25,26,27,30,32,33].

The capability of smartphones and smartwatches to monitor PD progression longitudinally was highlighted as particularly valuable. One study illustrated this potential by demonstrating significant changes in gait, tremor, finger tapping, and speech, tracked continuously over a period of 12 months. The study showed that these devices are capable of both active tasks (standardized assessments performed regularly by participants) and passive monitoring techniques (continuous accelerometry data collected via smartwatch), providing comprehensive longitudinal insights into symptom evolution. The integration of such continuous, high-resolution data collection allows clinicians to identify subtle, clinically meaningful variations in symptoms that may not be evident during periodic clinical visits [26]. Importantly, this longitudinal capability was supported by baseline validation, performed by their previous study, underscoring the practical utility of wearable technologies for tracking disease trajectories, informing timely adjustments in therapeutic interventions, and ultimately improving patient-centered care [27].

### 3.3. Usability in Clinical Practice

Usability and user acceptance are critical factors to ensure the facilitation of integration into routine care. Studies included highlighted variability in user compliance, engagement, and ease of use. One study reported an average adherence of 68.9% over a monitoring period of approximately 34 days, with participants completing an average of 2.7 daily tests, indicating a generally good level of sustained engagement with the smartphone-based protocol [23]. Promising usability and feasibility for a smartphone-based intervention was observed in an intervention aimed at increasing physical activity by counting steps, reflecting high perceived ease of use and overall positive feedback from participants [28]. Conversely, another study suggests that the usability of the mHealth application was limited, primarily due to low adherence rates, suggesting that the absence of direct clinical support and insufficient user-centered design might negatively impact patient engagement [25]. On the other hand, although usability was not explicitly examined as a primary outcome, one study reported a relatively good average engagement with their intervention, which also integrated a smartwatch-based step counter; their findings highlighted significant improvements in self-efficacy and non-motor symptoms. However, notably, there were no significant effects observed on motor symptoms [30]. Furthermore, a study reported that wrist-worn ATs were often perceived as more pleasant to use than hip-worn devices, suggesting a user preference for wrist-worn placement. These findings highlight the importance of ergonomic considerations and user interface accessibility, particularly in individuals with fine motor disabilities. Moreover, the majority of participants used the display at least once a day, underscoring the motivational potential of these devices when integrated into rehabilitation programs [32].

### 3.4. Accuracy of Commercial Activity Trackers

One study validated the accuracy of commercial ATs in measuring daily step counts, indicating good-to-excellent agreement with research-grade sensors. This underscores the potential for the widespread, cost-effective monitoring of physical activity levels, an important clinical target in PD management [32].

### 3.5. Differentiating PD from Healthy Subjects

Several included studies demonstrated that commercially available devices can discriminate individuals with PD from HSs. To assess the impact of PD on balance, gait, turn-to-sit, and sit-to-stand, a smartphone version of TUG was used in a study; findings evidenced how people with mild-to-moderate PD exhibit impaired postural control, altered gait strategy, and slower turn-to-sit performance than healthy subjects (HSs) [21]. One study reported over 95% accuracy in distinguishing PD participants from controls using a smartphone app that tracked voice, posture, and motor performance [23]. Significant differences were found between groups in gait parameters, which were captured with a smartphone-based gait analysis tool [22]. Additionally, smartphone-derived tactile cueing measures proved effective in differentiating PD and HSs, even under dual-task conditions [29]. Moreover, smartphone and smartwatch data identified characteristic PD motor features relative to controls [27,32]. Similarly, ATs were sufficiently sensitive to detect differences in daily activity levels between pwPD and HSs [32].

### 3.6. Improving Motor Performances

Tactile cueing (TC) delivered via a smartphone can improve the performance of complex tasks, even in the presence of a secondary motor task; this suggests that TC may be used to improve simple and complex motor performance in pwPD [29]. Also, even if another study focused on the feasibility and usability of an app encouraging physical activity, the findings reported an increase in daily step count in over 4 weeks [28]. In contrast, an intervention based on short text messages, information, and telephone counselling, had no effect on motor symptoms [30].

## 4. Risk of Bias

**Figure 2:**
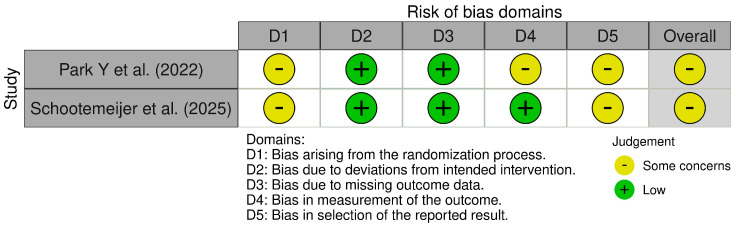
The risk of bias (RoB 2) regarding the use of smartphone apps [28,30].

**Figure 3:**
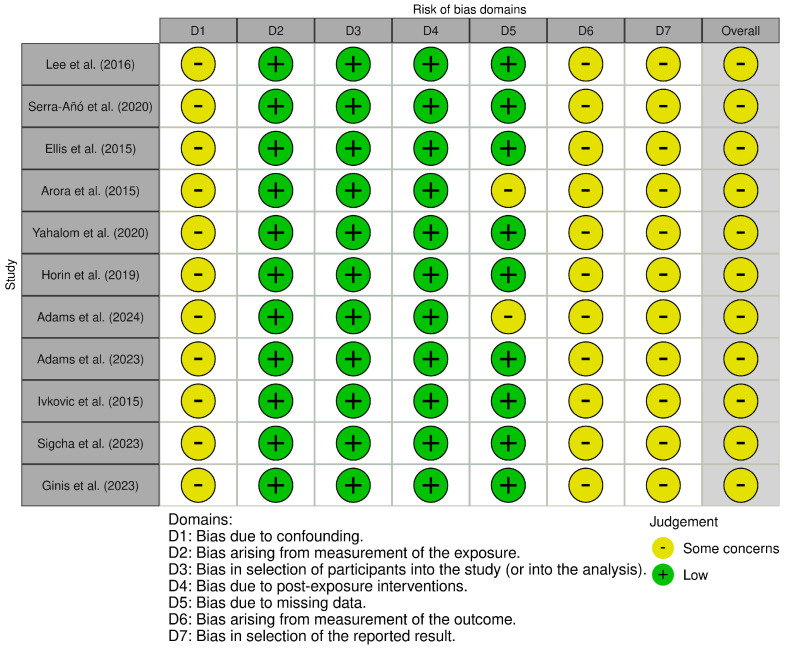
The risk of bias (ROBIN-E) regarding the use of smartphones, smartwatches, and Ats [20,21,22,23,24,25,26,27,29,31,32].

The Revised Cochrane Risk of Bias (RoB 2) tool [34] and the Cochrane Risk of Bias Assessment (ROBIN-E) tool [35] were used to assess the risk of bias of the articles included in this review; Figure 2 and Figure 3 summarize the assessments across the different domains. The RoB 2 tool was used for randomized control studies and presents five domains: “D1” bias arising from the randomization process, “D2” bias due to deviations from intended intervention, “D3” bias due to missing outcome data, “D4” bias in the measurement of the outcome, and “D5” bias in the selection of the reported result. The studies analyzed by the RoB 2 tool presented some concerns overall, primally due to limitations in the randomization process and selection of the reported result, while they presented a low risk in deviation from intended intervention and concerning missing outcome data. These issues may have introduced systematic differences at baseline or led to selective emphasis on positive findings, thus potentially overestimating intervention effects. The ROBIN-E tool was used for non-randomized controlled studies of exposure and presents seven domains: “D1” bias due to confounding variables, “D2” bias arising from the measurement of the exposure, “D3” bias in the selection of participants in the study (or in the analysis), “D4” bias due to post-exposure interventions, “D5” bias due to missing data, “D6” bias arising from the measurement of the outcome, and “D7” bias in the selection of the reported result. All observational studies assessed with the ROBIN-E tool were rated as having “some concerns” overall, particularly due to confounding variables, the measurement of the outcome, and the selection of the reported result. The lack of control for confounding variables may have affected the internal validity of the results, especially in studies assessing complex motor outcomes without baseline adjustment or stratification. Moreover, imprecise or unblinded outcome measurement could introduce detection bias, while the absence of pre-registered protocols raises the possibility of selective result inclusion. Domains concerning the selection of participants in the study, post-exposure interventions, and missing data showed low risk in general, suggesting robust methods and good validity in these specific aspects. Nevertheless, the identified biases must be considered when interpreting the findings of this review, particularly regarding effect sizes and generalizability. The overall strength of evidence should be regarded as moderate, warranting a cautious but constructive interpretation of the results.

## 5. Discussion

The interest in using wearable devices to manage health and mHealth intervention in chronic disease management is rapidly growing [36,37,38]. Devices such as smartphones and wrist-worn devices have revolutionized mobile health in the post-pandemic era, by enabling better disease prediction, prevention, diagnosis, and treatment [39].

The use of commercially available and relatively low-cost technologies in a widely studied disease such as Parkinson’s allows an overview of both active and passive measurement methods. Previous similar reviews have not only separately analyzed the use of smartwatches or smartphones and used assistive tools like inertial measurement units to assess gait [40] or non-commercial devices [18] but also analyzed their intervention in a wide range of diseases, such as movement disorders.

One of the major advantages of wearables is their global accessibility, which represents an important prerequisite for their successful integration into mHealth interventions: since their introduction in the last two decades, the use of smartphones has been increasing rapidly [41], and before COVID-19, global smartphone access had reached approximately 41.5% of the global population [42], representing a tool now rooted in the world population and accessible to all age groups. Smartwatches constitute an increasingly available category of wearable devices that can empower individuals to take care of their health right from their wrists [43]. Although, in relation to their functionalities, smartwatches may incur a higher cost compared to smartphones, which have advantages in biometric identification, due to their location on the wrist [44]. In contrast, smartphones require a strap or a similar tool to be attached to the body to achieve more accurate measurements.

As smartwatches, ATs are worn on the wrist; while smartwatches often include a touch screen, support advanced functionalities, and offer a high-resolution display, ATs are primarily designed for physical activity tracking. These characteristics, combined with the use of less expensive hardware and fewer sensors, makes them typically cheaper than smartwatches [45]. Despite their limited functionality compared to smartwatches, one study validated the accuracy of commercial ATs in measuring daily step counts in pwPD [32].

Digital data collection methods offer equivalent or superior time and cost efficiency, statistical reliability, and descriptive comparability compared to paper methods [46]. As demonstrated by several studies [20,21,22,23,24,27,31], there is a strong correlation between data from smartphone measurement and traditional clinical scales or assessment to evaluate PD. In addition to their clinical validity, another crucial factor is the usability of these technologies, which directly impacts adherence to treatment, especially in underdeveloped countries [47]. High usability is essential for user-centered mHealth applications targeting chronic illness populations, as it fosters sustained engagement, supports individualized care, and facilitates adherence to treatment and the successful integration of smartphones and smartwatches into routine clinical practice [33]. The studies included in this review revealed notable variability in user compliance, engagement, and perceived ease of use. Promising findings regarding usability emerged from a smartphone-based intervention designed to increase physical activity through step counting, demonstrating high perceived ease of use and positive participant feedback [28]. Although not primarily focused on usability, another study found a relatively good engagement level with a combined smartphone and smartwatch intervention, highlighting improvements predominantly in self-efficacy and non-motor symptoms rather than motor symptoms [30]. Conversely, another study found limited usability and adherence to an mHealth application, due to insufficient user-centered design and the absence of direct clinical support, underscoring how critical these aspects are for sustained patient engagement [25]. Furthermore, a study focusing on AT observed that wrist-worn activity trackers were generally preferred over hip-worn devices, particularly due to their greater perceived comfort and ease of use, emphasizing the importance of ergonomic considerations and user-friendly interfaces, especially among individuals with fine motor impairments. Moreover, a majority of participants interacted regularly with the activity tracker’s display, suggesting that providing immediate visual feedback can serve as a motivational tool within rehabilitation programs [32]. Collectively, these findings emphasize the need for carefully designed user interfaces and ergonomics, underscoring the critical role that devices can play in promoting adherence and optimal use, especially for individuals with more severe motor limitations. Future research should prioritize the collection of detailed patient feedback through structured usability assessments, employing standardized instruments or structured user interviews. Such assessments can offer valuable insights into the intuitiveness of device interfaces, ease of data interpretation, and the perceived utility of wearable devices in the everyday management of PD. Adopting a co-design methodology in collaboration with end-users and ensuring compatibility with existing clinical workflows, including integration with electronic health records and telemedicine platforms, will further facilitate usability and practical implementation in routine clinical care.

Wrist or hand-available technology may contribute to reducing hospital queues and healthcare costs [48] by limiting in-person consultations and enabling continuous monitoring. These technologies enable low-cost, on-demand measurements, saving patients’ money and time [49]. Additionally, mHealth technology could detect key motor and non-motor features of early, untreated PD, providing more reliable insights into symptom progression and intervention effectiveness, making it possible to track disease progression at a fraction of the cost of traditional monitoring methods [26,27]. In addition to their clinical applications, wearables have the potential to be effectively integrated into clinical practice, particularly in telemedicine, where data from these devices could be used to inform remote consultations and virtual check-ins with healthcare providers. Specific telemedicine applications, such as virtual consultations or remote monitoring programs leveraging wearable-generated data, could significantly enhance patient care by enabling clinicians to detect and address symptom fluctuations promptly. Personalized treatments informed by continuous wearable monitoring could optimize therapeutic efficacy, minimize in-person visits, and reduce healthcare system burdens, ultimately improving the quality of life for pwPD. As the adoption of telemedicine continues to grow, the synergy between wearable devices and remote healthcare services may provide patients with more accessible, personalized care. Furthermore, data collected by app could be used to train machine learning algorithms, as reported by a study for the automated assessment of postural instability enabled by smartphone [50], in which the machine learning approach was able to distinguish subjects with optimal, slightly impaired, and impaired postural control with high accuracy. In future, similar approaches could improve the ability of these tools to distinguish healthy from diseased individuals and have predictive models for complications such as fall risk. Several studies have already demonstrated the ability to distinguish HSs from pwPD [21,22,23,27,29,32], potentially making it possible to distinguish through an algorithmic approach both HSs and the staging of the disease.

To maximize the impact of wearable devices in PD management, future interventions should integrate principles from behavioral medicine. For example, the use of personalized and timely notifications or reminders delivered via a smartphone or smartwatch may help reinforce healthy routines, encourage physical activity, and support self-management behaviors in people with PD. When combined with real-time feedback from wearable sensors, these digital prompts can strengthen behavioral reinforcement mechanisms and promote sustained user engagement. Embedding such features into device design may enhance both usability and therapeutic efficacy, particularly in long-term monitoring contexts. Future research should also focus on refining these technologies to enhance usability, establishing standardized protocols for data collection, and conducting large-scale longitudinal studies to assess long-term benefits. Additionally, efforts should be made to explore the comparative effectiveness of different wearable devices, identifying which devices provide the best balance of affordability, accuracy, and user-friendliness for both patients and clinicians.

No study included in this review specifically analyzed the effectiveness of smartwatches as standalone devices. Smartwatches typically require pairing with a smartphone to function properly, and this could explain this absence. Variability in device placement and task protocols among studies may limit direct comparisons and the synthesis of findings, suggesting that future research should strive for standardized assessment methods. Furthermore, several studies showed substantial variability from pwPD, often including individuals at more advanced Hoehn & Yahr stages (>3); this variability could have influenced the effectiveness and generalizability of the findings.

Although the devices discussed in this review are portable and suitable for home-based monitoring, facilitating remote care for pwPD, many included studies were conducted in a clinical setting [20,21,22], while others did not clearly specify experimental settings [24,25,29,30,31], limiting our ability to confidently generalize these findings to everyday life scenarios. Moreover, some studies were short-term; thus, the findings may not accurately reflect long-term usage, adherence, or the natural variability of motor symptoms over time. Longitudinal studies would provide more reliable insights into symptom progression and the effectiveness of interventions. Additionally, the relatively small sample sizes of study participants may reduce the broader applicability of these results. Future studies should aim for larger, standardized task protocols and explicit evaluations of patient usability, acceptance, and barriers to technology adoption.

Smartphones, smartwatches, and AT offer valuable tools for assessing, monitoring, and improving motor symptoms in pwPD. The reviewed studies demonstrated many possible uses of this technology, like the ability to differentiate individuals with PD from HSs and the correlation between smartphone-data and established clinical measures. While smartphone-based methods showed promising usability, further research should address existing limitations, including standardized protocols, clearly defined study settings, and comparison between smartphones and wrist-worn devices. Leveraging these accessible, cost-effective technologies in clinical practice may enhance continuous monitoring, improve patient engagement, and support personalized disease management strategies, allowing a win–win condition both for patients and clinicians. These technologies can not only improve disease management through personalized interventions but also provide valuable data for clinicians, facilitating more proactive and cost-effective care. However, despite their promise, challenges related to standardization, device and data accuracy, and long-term usability and the need for user engagement remain. Future research should focus on overcoming these limitations, integrating wearables into clinical workflows, and conducting large-scale longitudinal studies to further validate their effectiveness. By addressing these gaps, wearable technologies can play a pivotal role in transforming the landscape of care for pwPD, offering benefits both to patients and healthcare providers.

## Figures and Tables

**Table 1:** Overview of main characteristics of involved studies.

Author	Study Design	Device	Aim	Population	H&Y	Intervention	Measure	Major Findings
Lee CY et al., 2016 [20]	Cross-sectional observational study	Smartphone, LG Optimus G	To develop a smartphone application to assess bradykinesia in PD.	57 PD, 87 HSs	1–3	Subjects were asked to alternately tap each side of the rectangles using an index finger at their fastest speed for ten seconds.	Number of correct tappings significantly correlated with motor UPDRS scores	Smartphone tapping application was comparable to conventional methods for the assessment of bradykinesia in PD.
Serra-Añó P. et al., 2020 [21]	Cross-sectional observational study	Smartphone, Xiaomi Redmi 4	To determine the impact of PD on motor symptoms using a single inertial measurement unit embedded in a smartphone device.	29 PD, 31 HSs	2–3	The device was attached just below the posterior superior iliac crests. Participants had to remain in a bipedal stance with their arms hanging relaxed alongside their body for 30 s. They had to perform the Time Up and Go (TUG) test.	Measure functional activities, such as balance, gait, turn-to-sit, and sit-to-stand. No comparison with other assessment.	People with mild- to moderate-stage PD display impaired postural control. Application distinguishes individuals with PD from HSs.
Ellis R.J. et al., 2015 [22]	Observational study	Smartphone, Apple iPod touch	To record gait movements during walking in PD.	12 PD, 12 HE	1.5–4	SmartMOVE utilizes the smartphone’s IMU to calculate successive step times and step lengths.	Outcome measures were validated against heel-mounted footswitches and a GAITRite sensor walkway while subjects walked along a prescribed path.	Smartphone-based gait analysis could be an alternative to conventional gait analysis methods.
Arora S. et al., 2015 [23]	Longitudinal observational study	Smartphone, LG Optimus S	To detect and monitor symptoms of PD.	10 PD, 10 HSs	N.D.	A smartphone application that assessed voice, posture, gait, finger tapping, and response time.	Measurement data extracted by the app were compared to UPDRS assessment.	Smartphones may represent an effective tool for the detection, assessment, and potentially care of PD.
Yahalom G. et al., 2020 [24]	Cross-sectional observational study	Smartphone, iPhone 6	To evaluate whether a smartphone-based TUG test can be used to identify postural instability in patients with PD.	44 PD, 22 HSs	N.D.	PD patients performed a 10-m TUG while motion sensor data were recorded from a smartphone attached to their sternum using the EncephaLog application.	Measurement data extracted by the app were compared to the UPDRS.	The use of smartphone motion sensors with applications may favor the early identification of patterns of gait dysfunction for different patient groups.
Horin A.P. et al., 2019 [25]	Prospective controlled study	Personal patient’s smartphone	To investigate the usability of a mobile health (mHealth) smartphone application to treat gait, speech, and dexterity in people with PD.	37 PD	2–3	Patients completed daily exercises for about 90 days, covering three domains: mobility, speech, and dexterity.	Gait parameters were measured using a walkway. Steps per day were measured by wearable sensors. The nine-hole peg test has been validated for use in PD and was used to assess dexterity. Speech measures were recorded using a microphone.	An mHealth application without therapist consultation or intervention was not adequate to improve gait, speech, or dexterity.
Adams, J.L. et al., 2024 [26]	Prospective multicenter observational study	Smartphone and smartwatch, Apple Watch 4 or 5, iPhone 10 or 11	To evaluate the longitudinal change in assessments of gait, tremor, finger tapping, and speech over 12 months.	82 PD, 50 HSs	1–5	The intervention consisted of cognitive, speech, and psychomotor tasks, tremor, gait, and balance tasks. During passive monitoring, accelerometry data and tremor scores were collected via a smartwatch.	Digital measurements were compared to MDS-UPDRS.	Digital assessments hold promise for helping evaluate the efficacy of future therapies and monitoring individuals in PD.
Adams, J.L. et al., 2023 [27]	Prospective multicenter observational study	Smartphone and smartwatch, Apple Watch 4 or 5, iPhone 10 or 11	To determine the specific disease features these digital tools can detect.	82 PD, 50 HSs	1–5	Participants wore the smartwatch on their more affected side and tracked symptoms on the smartphone daily for at least one week.	Digital measurements were compared to MDS-UPDRS.	A commercially available smartwatch and a smartphone research application can capture key motor and non-motor features of early, untreated PD.
Schootemeijer S. et al. 2025 [28]	Pilot double-blind randomized controlled trial	Patient’s personal smartphone	To investigate the feasibility and usability of a behavioral intervention using a motivational smartphone application to remotely increase physical activity in PD.	30 PD	1–3	An app to increase steps in PD through a motivational smartphone app by notifications and other feedback.	App usability was evaluated using the System Usability Scale and feature usage frequency, though no success criteria were predefined for the outcomes.	Titrated increase in daily step count is feasible over 4 weeks. The usability of the app was perceived as excellent.
Ivkovic V. et al., 2016 [29]	Crossover study design	Smartphone, MyTouch-3G™ HTC	To investigate the efficacy and limitations of tactile cueing (TC) for modulating simple and more complex motor tasks over a range of cueing intervals, with/without a secondary motor task.	10 PD, 10 HSs	2–4	To investigate the ability of PD patients and HSs to modulate simple heel tapping in response to TC. Investigated the ability of PD patients and HSs to modulate stepping during straight-line walking in response to TC.	No comparison with traditional evaluations or wearable sensors.	TC may be used to improve simple and complex motor performance in Parkinson’s disease patients.
Park Y. et al., 2022 [30]	Randomized clinical trial	Smartphone and smartwatch	To evaluate the effects of an mHealth intervention for self-management on self-efficacy, motor and non-motor symptoms, self-management, and quality of life in people with PD.	43 PD	Median 3	Mobile applications, smartwatches, smartphone-based short text messages. The control group received short text messages and telephone counselling for 16 weeks.	Motor symptoms of PD were measured using the UPDRS part III. For non-motor symptoms, the Non-Motor Symptoms Scale was used.	The mobile health intervention for self-management is effective for self-efficacy and non-motor symptoms in people with Parkinson’s disease.
Sigcha L et al., 2023 [31]	Prospective observational study	Smartphone and smartwatch, Tickwatch S2, Huawei Honor 9 Lite	To monitor motor symptoms of PD patients based on the performance of standardized exercises.	21 PD, 7 HSs	1–2.5	The wearable collected accelerometer data during each exercise and transferred it to the smartphone for analysis after all exercises were completed.	The specialist followed the MDS-UPDRS guidelines.	The proposed framework could be used as a complementary tool for the evaluation of motor symptoms in early-stage PD patients.
Ginis P. et al., 2023 [32]	Prospective observational study	Activity tracker (AT), FitBit Zip, FitBit Alta, FitBit Inspire	To investigate the validity of two commercial ATs to measure daily step counts.	28 PD, 30 HSs	N.D.	No specific instructions were given to monitor their step counts regularly during the use of the AT. PwPD wore the AT on their least affected side (wrist or hip). After the 14-day monitoring period, the devices were re-collected.	The ability to measure daily step fluctuations compared to a wearable sensor, which was previously validated in a laboratory setting for detecting step counts in pwPD compared to videotaped step counts.	ATs have sufficient criterion validity for daily use in early- to mid-stage PD.

Legend: PD = Parkinson’s disease; HSs = healthy subjects; H&Y = Hoehn & Yahr assessment; TUG = Time Up and Go; IMU = inertial measurement unit; UPDRS = Unified Parkinson’s Disease Rating Scale; mHealth = mobile health; TC = tactile cueing; ATs = activity trackers; pwPD = people with Parkinson’s disease.

**Table 2:** Summary table of study characteristics and reported findings.

Authors	Lee CY et al., 2016 [20]	Serra-Añó P. et al., 2020 [21]	Ellis R.J. et al., 2015 [22]	Arora S. et al., 2015 [23]	Yahalom G. et al., 2020 [24]	Horin A.P. et al., 2019 [25]	Adams, J.L. et al., 2024 [26]	Adams, J.L. et al., 2023 [27]	Schootemeijer S. et al. 2025 [28]	Ivkovic V. et al., 2016 [29]	Park Y. et al., 2022 [30]	Sigcha L et al., 2023 [31]	Ginis P. et al., 2023 [32]
**Correlation with Clinical Measures or wearable sensor and Ats accuracy**	The smartphone tapping application was comparable to the UPDRS for the assessment of bradykinesia in PD	n.a.	Smartmove was validated against heel-mounted footswitches and a GAITRite sensor walkway.	Smartphones were able to accurately estimate the severity of motor symptoms as measured by the UPDRS.	Smartphone-based TUG correlates with the UPDRS part II and III.	n.a.	n.a.	Smartphone and smartwatch measurements using the MDS-UPDRS scale have sometimes shown greater sensitivity in detecting some characteristics of PD compared to assessments based on traditional scales.	n.a.	n.a.	n.a.	Significant correlations between MDS-UPDRS and features extracted from the movement data used to assess resting tremor and bradykinesia.	The ATs’ accuracy was validated against Dynaport Movemonitor, a highly validated research-grade sensor.
**Monitoring of Symptoms and treatment**	n.a.	n.a.	n.a.	30 days	n.a.	90 days	365 days	365 days	21 days	n.a.	112 days	n.a.	14 days
**Usability in Clinical Practice**	n.a.	n.a.	n.a.	Participants showed high adherence rates.	n.a.	Participants showed low adherence rates.	n.a.	n.a.	High perceived usability and positive feedback from participants.	n.a.	Usability was not assessed as the primary outcome, but moderate involvement was found.	n.a.	Usability influenced by ergonomics. A total of 79% of participants used the display every day, indicating its motivational potential.
**Differentiating PD from Healthy Subjects**	n.a.	Significant differences in balance, gait strategy, and turn-to-sit transition between PD and controls.	Significant differences in gait parameters between PD and HSs.	High accuracy in discriminating between PD and HSs.	n.a.	n.a.	n.a.	Smartphones and smartwatches detected motor patterns characteristic of PD compared to controls.	n.a.	Tactile cueing via smartphone effectively differentiated PD and HSs even under dual-task conditions.	n.a.	n.a.	ATs found significant differences in daily activity levels between PD and HSs.
**Improving motor performances**	n.a.	n.a.	n.a.	n.a.	n.a.	n.a.	n.a.	n.a.	Increased daily steps in 4 weeks via motivational app.	Tactile cueing via a smartphone improved motor performance even under dual-task conditions.	Intervention with messages, information, and telephone calls did not improve motor symptoms.	n.a.	n.a.

Legend: n.a. = not applicable; UPDRS = Unified Parkinson’s Disease Rating Scale; PD = Parkinson’s disease; TUG = Time Up and Go; AT = activity tracker; HSs = healthy subjects.

## Data Availability

Not applicable.

## Abbreviations

The following abbreviations are used in this manuscript:

PD	Parkinson’s Disease
AT	Activity tracker
pwPD	People with Parkinson’s disease
HSs	Healthy subjects
H&Y	Hoehn and Yahr assessment
TUG	Time Up and Go
IMU	Inertial measurement unit
UPDRS	Unified Parkinson’s Disease Rating Scale
mHealth	Mobile health
TC	Tactile cueing
